# Exploring phylogenetic relationships within the subgenera of *Bambusa* based on DNA barcodes and morphological characteristics

**DOI:** 10.1038/s41598-022-12094-8

**Published:** 2022-05-16

**Authors:** An Ke Wang, Qi Fan Lu, Zhen Xian Zhu, Sheng Hui Liu, Hao Zhong, Zi Zhang Xiao, Yue Guo Zou, Li Jian Gu, Xu Hua Du, Han Jiang Cai, Yu Fang Bi

**Affiliations:** 1 Key Laboratory of Resources and Utilization of Bamboo of State Forestry Administration, China National Bamboo Research Center, Hangzhou, 310012 Zhejiang China; 2 Monitoring Center for Forest Resources in Zhejiang Province, Hangzhou, 310020 Zhejiang China; 3 Forestry Bureau of Hua’an, Zhangzhou, 363000 Fujian China; 4 Hangzhou Lin’an Taihuyuan Ornamental Bamboo Planting Garden, Hangzhou, 310020 Zhejiang China

**Keywords:** Taxonomy, DNA sequencing

## Abstract

The genus *Bambusa* belongs to the subtribe Bambusinae and the subfamily Bambusoideae. The subgenera of *Bambusa* has not been satisfactorily circumscribed, and this remains a major taxonomic issue. Simultaneously, genera such as *Dendrocalamus* and *Gigantochloa* have not been confidently assigned to *Bambusa*. Here, the phylogenetic relationships among subgenera were investigated using five chloroplast DNA markers (rpl32-trnL, rpl16, matK, rbcL, and trnH-psbA) for a sample of 50 ingroup and 16 outgroup species. A total of 186 key morphological descriptors were studied for the 50 ingroup species. The results indicated that five chloroplast DNA markers were possible to distinguish *Bambusa* species from other species and divide them into several clusters. Phylogenetic analyses conducted using morphological descriptors and a combined marker (rpl32-trnL+rpl16) revealed three and five distinct lineages, respectively, among the currently recognized *Bambusa* species. The branching pattern of the dendrogram was not completely consistent with the classical taxonomic classification of *Bambusa*. In addition, not all varieties and cultivars were clustered with McClure classifications. As the maximum parsimony topology and morphological analyses were inconsistent, some clustering results overlapped. Overall, the results obtained here do not support the current classification of the *Bambusa* subgenera.

## Introduction

The genus *Bambusa*, belonging to the subtribe Bambusinae and the subfamily Bambusoideae, is among the largest woody bamboo genera, comprising over 100 species^1^. The classical botanical classification of bamboo species is based on the morphological characteristics of their culms, branches, and sheaths, owing to the infrequency of their blossoming. The Flora of China^2^ (FOC), the newest authoritative botany book in China, divides *Bambusa* into four subgenera based on the morphological characteristics of the culm, branch, and sheath: *Bambusa*, *Leleba*, *Lingnania*, and *Dendrocalamopsis*. The subgenus *Dendrocalamopsis* was not included in previous versions of the Flora Reipublicae Popularis Sinicae^3^ (FRPS). The FOC has been updated to reflect new findings in bamboo research; however, the definitions of some species remain controversial. Like *Bambusa oldhamii*, some studies continue to use the former name *Dendrocalamopsis oldhamii*^4^.

The distinctive life pattern, such as infrequent flowering and predominance of asexual reproduction render the taxonomic classification of bamboo difficult^5^. Consequently, there have been many misnamed species. For example, the *Bambusa* species *B. chungii*, *B. guangxiensis*, and *B. cerosissima* have been misnamed *Lingnania chungii*, *L. funghomii,* and *L. cerosissima*, respectively^2^. With the addition of flowering materials, Chinese species were hitherto placed in the *Sasa* subgenus *Sasa*. Qin^6^ et al. strongly argued that, considering the monophyly of the Chinese representatives of the *Sasa* subgenus *Sasa,* a new genus, *Sinosasa,* should be erected.

Both morphological and molecular systematics have been utilized to solve the problems of bamboo classification. Among the various approaches used in molecular systematics, DNA sequencing has become one of the most widely used methods applied in bamboo classification^7,8^, especially at the genus level^9,10^. Recently, substantial progress has been made towards understanding the evolutionary relationships of *Bambusa* and its allies (*Bambusa*, *Dendrocalamus*, *Gigantochloa*, and *Melocalamus* are classified as a close group, in particular based on their shared characteristic of a solid, thickened, and hairy ovary summit) using molecular data^11^. Yang^11^ et al. used nuclear gene (GBSSI) and plastid DNA sequences (psbA-trnH, rpl32-trnL, and rps16), which allowed *Bambusa* and *Dendrocalamopsis* to be classified into one of two clades with reasonable support. Through this approach, 17 *Bambusa* samples were classified into three clades, and this result supported the present subgeneric classification of *Bambusa*. However, other studies have not supported this classification. The phylogeny of bamboo species has also been analyzed using only internal transcribed spacer (ITS) sequences. In this group, each branch was composed of several species of three subgenera (not including the subgenus *Dendrocalamopsis*), and the *Bambusa* and *Dendrocalamus* species formed a group with a bootstrap value of 100^12^. Goh^13,14^ et al. used chloroplast DNA markers (rps16-trnQ, trnC-rpoB, and trnD-T) and a nuclear DNA marker (GBSSI) to classify *Bambusa*, *Dendrocalamus*, and *Gigantochloa* as distinct lineages. This approach identified four *Bambusa* subgenera, which differed from the subgeneric classification. Chloroplast DNA sequences have been extensively used to infer plant phylogeny for uniparental inheritance through comparison with nuclear DNA sequences^15^.

Several DNA markers have been used as core plant barcodes, such as the plastid (chloroplast) markers rbcL, matK, and trnH-psbA. Nuclear ribosomal ITSs have also been used^16^. Statistical results revealed that these three plastid markers showed high levels of universality (87.1–92.7%) and that the combination of ITS and any of the plastid DNA markers was able to discriminate 69.9–79.1% of species^17^. In this study, many DNA barcoding primers (trnL-trnF, trnS-trnG, psbB-psbF, rpl16, rpl32-trnL, rbcL, matK, trnH-psbA, and ITS) were utilized with the aim of amplifying the DNA sequences of bamboo samples. Unfortunately, the plastid DNA markers trnL-trnF, trnS-trnG, and psbB-psbF failed to amplify most specimens, as did the nuclear marker ITS.

In addition to DNA barcoding, researchers have attempted to arrange morphological characteristics into a data matrix using cladistic analysis^18^. DAS^19^ et al. scored 32 key morphological descriptors for 15 bamboo species and standardized them as qualitative and quantitative interval data to construct a tree graph, using the unweighted pair-group method of arithmetic averages. For other plants, Tilney^20^ et al*.* used morphological and anatomical characteristics as scoring feature matrices for the cladistic analysis of *Lichtensteinia* (Apiaceae). Based on morphological data, Kim^21^ et al*.* conducted principal component analysis and cluster analysis on native chrysanthemum in South Korea.

In the present study, the phylogenetic relationships among the four subgenera of *Bambusa* were investigated (50 samples) using DNA sequence data and morphological characteristics, employing a much larger taxon sample than has been previously available. This included representatives from all subgenera of *Bambusa* that have previously been described. DNA sequence data were derived from the plastid markers.

## Materials and methods

### Materials

A total of 66 taxa from *Bambusa* and some other bamboo species (Table 1, all Latin names were obtained from the FOC) representing ten genera were sampled for molecular phylogenetic analysis. There were 50 species from *Bambusa* belonging to the four subgenera described in the FOC^2^, including *D. oldhamii* and *Neosinocalamus affinis*, which were not accepted as *Bambusa* in the FRPS, but were moved to *Bambusa* in 2007^2^ and named *B. oldhamii* and *B. emeiensis*, respectively. The outgroup taxa included *Dendrocalamus*, *Drepanostachyum*, *Indosasa*, *Melocanna*, *Neosinocalamus*, *Oligostachyum*, *Phyllostachys*, *Pseudosasa*, *Pleioblastus*, *Shibataea*, and *Sinobambusa*. Fifty taxa from *Bambusa* were collected and analyzed for morphological phylogeny.

**Table 1:** Sixty-six taxa from *Bambusa* and the outgroup.

Name of bamboo	Missing data	Name of bamboo	Missing data
*Bambusa albolineata*		*Bambusa pachinensis*	
*Bambusa arundinacea*		*Bambusa pachinensis* var. *hirsutissima*	
*Bambusa blumeana*		*Bambusa pervariabilis*	
*Bambusa boniopsis*		*Bambusa prominens*	
*Bambusa cerosissima*		*Bambusa sinospinosa*	
*Bambusa chungii*		*Bambusa surrecta*	
*Bambusa chungii* var. *velutina*		*Bambusa teres*	
*Bambusa cornigera*		*Bambusa textilis*	
*Bambusa contracta*		*Bambusa textilis* var. *gracilis*	
*Bambusa corniculata*		*Bambusa textilis* cv. Purpurascens	
*Bambusa distegia*		*Bambusa tuldoides*	
*Bambusa dolichoclada*	trnH-psbA	*Bambusa tuldoides* cv. Swolleninternode	
*Bambusa duriuscula*	rpl16	*Bambusa ventricosa* cv. Nana	
*Bambusa emeiensis / N. affinis*		*Bambusa vulgaris*	
*Bambusa eutuldoides*		*Bambusa vulgaris* cv. Vittata	
*Bambusa eutuldoides* var. *basistriata*		*Bambusa vulgaris* cv. Wamin	
*Bambusa eutuldoides* var. *viridi-vittata*		*Bambusa xiashanensis*	
*Bambusa flexuosa*		*Dendrocalamus membranaceus*	*
*Bambusa gibba*		*Dendrocalamus minor*	*rpl16
*Bambusa gibboides*	rpl32-trnL, rpl16	*Dendrocalamus minor* var. *amoenus*	*
*Bambusa indigena*		*Drepanostachyum scandens*	*
*Bambusa lenta*		*Indosasa shibataeoides*	*matK
*Bambusa longispiculata*		*Melocanna baccifera*	*
*Bambusa macrotis*		*Neosinocalamus affinis* cv*.* Viridiflavus	*trnH-psbA, rpl16
*Bambusa multiplex*		*Oligostachyum lubricum*	*rbcL
*Bambusa multiplex* cv. Alphonse-Karr		*Phyllostachys heteroclada*	*matK
*Bambusa multiplex* cv. Fernleaf		*Phyllostachys heterocycla*	*
*Bambusa multiplex* cv. Silverstripe		*Phyllostachys violascens*	*
*Bambusa multiplex* cv. Stripestem Fernleaf		*Pseudosasa amabilis*	*rbcL
*Bambusa multiplex* var. *riviereorum*		*Pseudosasa japonica* var. *tsutsumiana*	*
*Bambusa multiplex* var. *shimadae*		*Pleioblastus viridistriatus*	*
*Bambusa mutabilis*		*Shibataea chinensis* cv. Aureo-striata	*trnH-psbA
*Bambusa oldhamii / D. oldhamii*	*	*Sinobambusa tootsik* var. *luteolo-albo-striata*	*

Marker name in the missing data column indicates that there was an amplification or sequencing failure; * indicates missing morphological characteristic data.

### DNA isolation, amplification, cloning, and sequencing

Leaves were collected from the Hua’an Bamboo Garden (Fujian Province, China) and Lin’an Taihu Lake Source Bamboo Garden (Zhejiang Province, China). Total DNA was extracted from silica-gel-dried young leaves, using a modification of the method described by Fulton^22^ et al. Polymerase chain reaction (PCR) amplification, cloning, and the sequencing of rpl16 were performed according to the forward^23^ and reverse primers^24^, following the protocol of Cornelia^23^ et al*.* For rpl32-trnL, the primers rpl32-F and trnL were used, following the protocol of Shaw^25^ et al.; for rbcL, the primers rbcL-1F and rbcL-724R were used, following the protocol of Fay^26^ et al.; for matK, the primers matK-ML and matK-MU were used, following the protocol of Zhu^27^ et al*.*; and for the psbA-trnH region, the primers psbA^28^ and trnH2^29^ were used, in accordance with the protocol of Tate and Simpson^30^. All the primer sequences are shown in Table 2.

**Table 2:** Sequences of the five primers used in this study.

Mark	Prime-F	Prime-R
rpl32-trnL	CTGCTTCCTAAGAGCAGCGT	CAGTTCCAAAAAAACGTACTTC
rpl16	CTATGCTTAGTGTGTGACTC	TCTTCCTCTATGTTGTTTACG
matK	AAACAGAAATCTCGTCAA	AGGGTTCACCAGGTCATT
rbcL	ATGTCACCACAAACAGAGACTAAAGC	TCGCATGTACCTGCAGTAGC
trnH-psbA	CGCGCATGGTGGATTCACAATCC	GTTATGCATGAACGTAATGCTC

PCR was conducted using the TaKaRa Ex™ kit (Takara Biomedical Technology Co., Ltd., Beijing, China) with the following program settings: 5 min at 95.0 °C; 35 cycles of 30 s at 95.0 °C, 30 s at annealing temperature, 40 s at 72.0 °C; 7 min at 72.0 °C; and then holding at 4.0 °C. The annealing temperatures used here were 51.0–56.0 °C. The PCR reaction mixture contained 10 ng of DNA samples, 0.5 μL (10 μM) each of forward and reverse primers, 0.5 μL of deoxyribonucleotide triphosphate (dNTP), 2.5 μL of 10 × buffer, and 0.5 μL of deoxyribonuclease (DNase); double distilled water (ddH_2_O) was added to make the volume up to 25 μL. PCR products were purified using Promega Wizard® PCR Clean-up System kits (Promega Biotech Co., Ltd., Beijing, China) following the manufacturer’s instructions. DNA sequencing was performed commercially by Shanghai Sunny Biotechnology Co., Ltd. (Shanghai, China).

### DNA sequence alignment and phylogenetic analyses

DNA sequences were edited using CHROMAS v2.6.5 and aligned by MUSCLE (embedded in MEGAX), with default parameters. They were adjusted manually where necessary. All sequence data were uploaded to the National Center for Biotechnology Information (NCBI) (https://www.ncbi.nlm.nih.gov/Traces/study/?acc=PRJNA706162&o=acc_s%3Aa). Maximum parsimony (MP) analysis was conducted based on the separate rpl32-trnL, rpl16, matK, rbcL, and trnH-psbA datasets and with a combined rpl32-trnL+rpl16 dataset.

MP analysis was performed with MEGAX (https://www.megasoftware.net/); all characteristics were equally weighted, and gaps were coded as missing data. Heuristic searches of 1,000 random addition replicates were conducted using subtree-pruning-regrafting (SPR) branch swapping. This was done to obtain the most parsimonious trees, and ten trees from each random sequence were saved. Estimates of clade robustness were obtained through bootstrap values (BV) calculated from 1000 replicate analyses, conducted using the heuristic search strategy and through a simple addition sequence of the taxa. The incongruence length difference (ILD) test of Farris^31^ et al. was used to evaluate the statistical significance of character incongruence among the rpl32-trnL and rpl16 intron datasets before their combined analysis.

### Morphological characteristic analyses

Based on the China Industry Standard Guidelines for conducting distinctness, uniformity, and stability tests, 186 key morphological descriptors were used to assess *Bambusa* members. Morphological descriptors were scored as follows: each species was considered as a separate independent operational taxonomic unit (OTU). One hundred and eighty-six key morphological descriptors were used (one root descriptor about aerial root; 22 culm descriptors about powder ring, hair ring, surface cover, color, internode length, diameter, shape, and sheath-node bulge; nine branch descriptors about branch thorn, lowest branch height, and leaf number; six leaf descriptors for length, hair, and base shape; 18 culm descriptors for sheaths about surface cover, hair ring, brim hair, length and color streak; 50 descriptors for sheath auricles about length, the length ratio value of the two auricles, corrugated fold, shape, oral setae length, root location, and extension condition; 54 sheath blade descriptors about shape, reflex, corrugation, hairy, color, tip shape, base length, and length; and 26 sheath ligule descriptors about length, shape, eyelash, and eyelash length). The specific morphological characteristics that were selected are listed in Table A1, which were assessed from each of the 50 OTUs (five replications per OTU) studied in the field. Mean values obtained from five independent replications were used as representative OTU data for each quantitative morphological descriptor. If the sample characteristics conformed to descriptors, they were marked as “0”; if not, they were marked as “1.” The scored qualitative and quantitative interval data were standardized to construct a dendrogram using neighbor-joining (NJ) performed via PowerMarker V3.25.

### Ethical approval

This article does not contain any studies with human participants or animals performed by any of the authors.

### Herbarium vouchers

Hua'an Bamboo Botanical Garden and Hangzhou Lin’an Taihuyuan Ornamental Bamboo Planting Garden supported the research work of this article. The Materials were collected by Y.G.Z and L.J.G in accordance with related management rules without damaging the growth of bamboo, and the relevant herbarium vouchers were kept in Hua'an botanical garden and Hangzhou Lin’an Taihuyuan Ornamental Bamboo Planting Garden. The list of specific species is attached in the annex (Table A2).

## Results

### Phylogenetic analyses

In this study, all five markers, rpl32-trnL, rpl16, matK, rbcL, and trnH-psbA, were independently detected by MP. Based on the five MP trees and supplemented by information on diversity acquired using DnaSP v5^32^, phylogenetic analyses of *Bambusa* were performed using combined DNA barcoding (rpl32-trnL+rpl16).

The nucleotide (Pi) and haplotype (Hd) diversities of these five DNA barcodes indicated that rbcL and trnH-psbA were not suitable for identifying *Bambusa* as their Pi values were much lower than those of rpl 32-trnL, rpl 16, and matK at 0.00458 and 0.00406, respectively (Table 3). In contrast, rpl32-trnL, rp16, and matK appeared to be reasonable barcoding candidates for identifying *Bambusa* species according to the diversity information available. While matK, rbcL, and trnH-psbA could separate *Bambusa* from other genera, they grouped >70% of the sampled *Bambusa* into one cluster. In comparison, rpl32-trnL and rpl16 both divided *Bambusa* into several clusters.

**Table 3:** Diversity information of DNA sites based on 66 bamboo species.

Mark	Number of the sites	Nucleotide diversity, Pi	Haplotype diversity, Hd
rpl32-trnL	1109	0.07872	0.903
rpl16	1283	0.07573	0.771
matK	1611	0.06624	0.803
rbcL	698	0.00458	0.587
trnH-psbA	636	0.00406	0.581
rpl16 + rpl32-trnL	2392	0.08462	0.961

**Figure 1:**
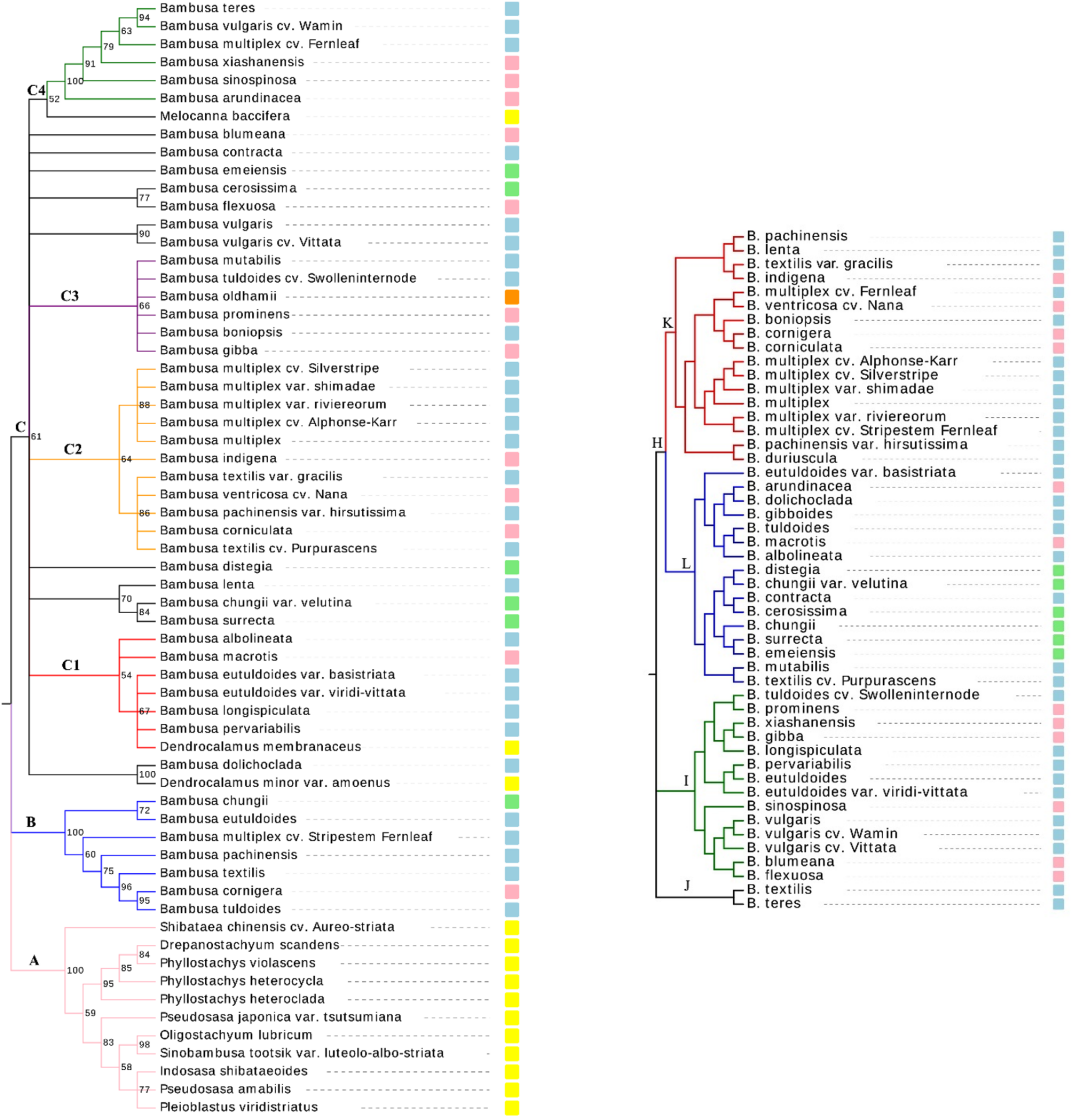
Strict consensus of the most parsimonious trees based on two cpDNA datasets (left), dendrogram derived from NJ cluster analysis based on 186 morphological descriptors of 50 bamboo species (right). Strips with different colors indicate subgenera: *Lingnania* (blue), *Bambusa* (pink), *Leleba* (green), *Dendrocalamopsis* (orange), and the outgroup (yellow).

A combined barcode (rpl 32-trnL+rpl 16) was also used to analyze the phylogeny of 66 taxa after the ILD was tested. The *p*-value of the ILD was 0.05 and the combined marker successfully divided bamboo into several clusters, as shown in Fig. 1 (left). The tree length, consistency index (CI), and retention index (RI) of the MP analyses for rpl32-trnL+rpl16 were 2392, 0.79, and 0.91, respectively. The BVs were mapped onto the MP topologies and shown as figures behind the branches. The analysis based on the rpl32-trnL+rpl16 combined dataset divided the entire group into three major clusters (A [100 BV], B [100 BV], C [61 BV]), with cluster A as an outgroup (Fig. 1, left), constituting members of the *Shibataea, Drepanostachyum*. *Phyllostachys*, *Pseudosasa*, *Oligostachyum*, *Sinobambusa*, *Indosasa*, and *Pleioblastus*. However, three species, *Dendrocalamus minor* var. *amoenus*, *Dendrocalamus membranaceus*, and *Melocanna baccifera* (cluster C) were considered to form an outgroup and were therefore not included. *Bambusa* taxa formed two major clusters, B and C, and cluster C was further divided into four sub-clusters (C1 [54 BV], C2 [64 BV], C3 [66 BV], and C4 [52 BV]) and several monotypic and small clades.

The branching pattern of the dendrogram was not completely consistent with the classical taxonomic classification of *Bambusa* proposed by the FOC^2^, especially at the subgenus level. The subgenus *Lingnania* (blue strip in Fig. 1) contained the greatest number of species sampled in this study, while members the subgenus *Bambusa* (pink strip) were scattered among all the *Bambusa* clusters. The subgenus *Leleba* (green strip) did not appear in the four sub-clusters of cluster C, and essentially was found in several monotypic and small clades of cluster C, except for *B. chungii* in cluster B. *B. oldhamii* (orange stripe) belonged to the subgenus *Dendrocalamopsis* in cluster C3. Meanwhile, several species belonging to the same subgenus were also clustered with a high degree of confidence in our MP analysis. For example, in cluster C2, most of the species belonged to the subgenus *Leleba*, except for *B. contracta*, *B. ventricosa* cv. Nana, and *B. indigena*, while in cluster C1, most species belonged to the subgenus *Leleba*, except *for B. macrotis* and *D. membranaceus*.

Varieties and cultivars did not always stay with their McClure classifications. For example, *B. chungii* and *B. chungii* var. *velutina* were separated into two clusters and two varieties of *B. eutuldoides* were assigned to cluster C1 but were separated from *B. eutuldoides* (in B). Meanwhile, cultivars of *B. textilis* were assigned to cluster C2 but were separated from *B. textilis* (in B), and *B. vulgaris* and *B. vulgari*s cv. Vittata formed a small cluster (90 BV) but were separated from *B. vulgaris* cv. Wamin. *Bambusa multiplex* and its varieties and cultivars, except for *B. multiplex* cv. Fernleaf and *B. multiplex* cv. Stripestem Fernleaf, formed a sub-cluster of cluster C2 with 88 BV.

### Analyses of morphological characteristics

In the absence of flower or fruit characteristics, culm sheaths and characteristics were treated as two taxonomic features for classifying *Bambusa*. According to 186 key morphological descriptors, the entire dendrogram (Fig. 1, right) was split into three clusters (H, I, and J). One main cluster (H) was divided into two sub-clusters (K and L) and four clusters (K, L, I, and J) did not completely conform to the existing classification. For instance, *B. textilis* and *B. teres* were totally isolated in a small cluster (J) belonging to the subgenus *Leleba* and species in the subgenus *Lingnania* were all in one subclade of cluster L. Critically, the subgenera *Bambusa* and *Leleba* were not separated from one another. Meanwhile, varieties and cultivars, such as *B. vulgaris* and *B. multiplex*, were more likely to stay with their McClure classifications. *Bambusa vulgaris* and its two cultivars formed a small clade in cluster I and *B. chungii* and *B. chungii* var. *velutina* were grouped into cluster L. The varieties and cultivars of *B. multiplex* were placed into cluster K; *B. textilis*, *B. textilis* cv. Purpurascens, and *B. textilis* var. *gracilis* were split into clusters J, L, and K, respectively, and *B. tuldoides* cv. Swolleninternode and *B. tuldoides* were split into clusters I and L.

### Topological congruences

The MP topology analyses were largely inconsistent with the morphological analysis, but cluster C2 in the MP analysis was largely consistent with cluster K in the morphological analysis. This highly consistent cluster included *B. textilis* var. gracilis, *B. indigena*, *B. pachinensis* var. *hirsutissima*, *B. ventricosa* cv. Nana, *B. corniculata*, *B. multiplex*, *B. multiplex* cv. Alphonse-Karr, *B. multiplex* cv. Silverstripe, *B. multiplex* var. *shimadai*, and *B. multiplex* var. *riviereorum*. These species share some of the same characteristics: no aerial root, wedge-shaped leaf base, sheath clade length/culm sheath length < 1, sheath blade erect, and hairy ventral.

## Discussion

### *Bambusa* and its allies

In the present study, the DNA barcode rpl32-trnL+rpl16 identified *Bambusa* from other genera that were close to *Bambusa*, although it struggled to distinguish *Bambusa* from *Dendrocalamus*. Previous molecular studies have not convincingly shown that *Bambusa* is a monophyletic genus when related genera have also been considered. Sun^12^ et al. used ITS and random amplified polymorphic DNA (RAPD) and found that three *Dendrocalamus* species (*D. latiflorus*, *D. membranaceus*, and *D. strictus*) were nested among the *Bambusa* taxa. Yang^33^ et al. used the combined ITS+GBSSI+trnL-F combinatorial regions to show that eight *Bambusa* taxa (including *B. oldhamii*) were resolved as a monotypic clade in a phylogenetic tree supported by the posterior probability of Bayesian analysis. However, the sister grouping of *Dendrocalamus* has been strongly supported. Goh^14^ et al*.* used the combined plastid DNA rps16-trnQ+trnC-rpoB+trnD-T and sampled 53 kinds of bamboo. They determined that *Dendrocalamus* and *Gigantochloa* were embedded in *Bambusa* taxa; however, the nuclear DNA marker (GBSSI) indicated that *Dendrocalamus* may exist as a subclade departed from *Bambusa*, but can still be considered its sister. DAS^19^ et al. attempted to construct a phylogenetic tree using 32 morphological characteristics for 15 bamboo species, but failed to separate *Bambusa*, *Dendrocalamus*, and *Gigantochloa* successfully. Here, *Dendrocalamus* was completely embedded in *Bambusa* taxa. *B. emeiensis* and *B. oldhamii* were also intermixed with *Bambusa*; they were classified as new members of *Bambusa*, having previously been named *N. affinis* and *D. oldhamii*, respectively.

### Morphological characteristics analyses and subgeneric classification

According to the FOC, the genus *Bambusa* has four subgenera: *Lingnania*, *Dendrocalamopsis*, *Bambusa*, and *Leleba*. The subgenus *Lingnania* was found to share the following typical characteristics: a culm sheath with a narrow blade, a base only one-third of the width of the sheath apex; culm internodes that are usually longer than 30 cm, and thin walls (often < 8 mm). Three other subgenera shared the following characteristics: a culm sheath with a broad blade, a base 1/2–3/4 of the width of the sheath apex; culm internodes shorter than 30 cm, and thick walls (up to 2 cm). Meanwhile, the subgenus *Dendrocalamopsis* shared the following typical characteristics: culm sheath auricles and small, and rounded spikelets that are dense at maturity. The rest of the subgenera shared the following characteristics: culm sheath auricles that are large, rounded, irregular, or absent and spikelets that are loose at maturity, with broad florets on short rachilla segments. Otherwise, the characteristics of the subgenus *Bambusa* were found to be branchlets of lower branches specialized into tough or weak leafless thorns, and with culm sheaths with persistent blades. The subgenus *Leleba* had branchlets in their lower branches that were normal and leafy; and their culm sheath blade was deciduous.

To the best of our knowledge, this study represents the first attempt to distinguish *Bambusa* subgenera by using 186 morphological descriptors to sample more than 50 *Bambusa* taxa. As mentioned above, the traditional classification uses eight to 14 morphological characteristics to identify a subgenus, which are fewer than the number of morphological characters used in this study. Therefore, it is not surprising that the morphological phylogenetic tree generated here did not coincide exactly with the existing *Bambusa* subgenus classification. Establishing a phylogenetic tree based on morphological characteristics is a novel way to explore bamboo classification. According to the findings of this approach, we described more than 39 morphological features as 186 key morphological descriptors. Thus, the results were focused more on the overall characteristics of each species, rather than on one or several obvious or easily identifiable features.

### Controversial bamboo species

The FRPS classified *B. arundinacea* as a member of the subgenus *Bambusa*. However, Xia^2^ et al. pointed out that *B. vulgaris* was incorrectly named by Aiton as *B. arundinacea* and that *B. auriculata* and *B. striata* were also the same species as *B. vulgaris*. DAS^19^ et al. did not support this point based on morphological characters and molecular analysis. Instead, they found that, from a morphological perspective, these four bamboo species (*B. arundinacea*, *B. vulgaris*, *B. auriculata*, and *B. striata*) differed from each other, and *B. striata* and *B. vulgaris* showed greater similarity to each other than the others in RAPD analysis. Here, *B. auriculata* and *B. striata* were not sampled, and the data of morphological characteristics and DNA sequence between *B. arundinacea* and *B. vulgaris* were different in this study.

*B. chungii* var. *velutina* is a new variant of *B. chungii* that, to date, has only been found in the Fujian province of China. It was previously considered as a member of the genus *Lingnania*, but is now considered to be a sub-genus of *Bambusa*. Here, *B. chungii* and *B. chungii* var. *velutina* were found to be similar in both MP and morphological characteristic analyses.

### Application of the codes

Compared with flowering plants, the classification of bamboos is more challenging for researchers and workers that are not engaged in examining phylogenetic relationships. Using DNA barcodes to classify or identify species will be more widely applied with the growth of molecular biology technology because of its easy operability, even though it may not align perfectly with traditional botanical classification. The codes rpl32-trnL and rpl16 are two loci on plastid DNA. Phylogenetic analyses that are based on whole chloroplast genomes have been used to resolve relationships within the subfamily Bambusoideae^5^. Wang^34^ et al. suggested the use of a larger dataset, indicating that insufficient parsimony information characters were the main cause for poor resolution in temperate bamboos.

Based on morphological features, morphological codes were used as a classification method to evaluate whether they could be a match for traditional classification. However, following statistical analysis, results showed that it could not be appropriately explained in the context of morphological classification. A new operating model for morphological codes needs to be developed for the application of this technique in botanical classification.

## Supplementary Information

Supplementary Information 1.

Supplementary Information 2.
