# Supplementary material for: Exploring phylogenetic relationships within the subgenera of Bambusa based on DNA barcodes and morphological characteristics
Source: Sci Rep. 2022 May 16;12:8018. doi: 10.1038/s41598-022-12094-8 (PMC9110708; doi:10.1038/s41598-022-12094-8)
Supplement: Supplementary file 1 — Supplementary Information 1. [file 41598_2022_12094_MOESM1_ESM.docx]

Table A1. Morphological descriptors.

| **Organ** | **Characteristics** | **Comments** | **Representative species** | |
| --- | --- | --- | --- | --- |
|  |  |  | Match description(0) | Not match description(1) |
| Root | Aerial root |  | N/A | *B. emeiensis / N. affinis* |
| Culm (infancy) | With a ring of white powder below sheath scars |  | *B. pachinensis* | *B. blumeana* |
|  | Covered with white powder |  | *B. contracta* | *B. multiplex* cv. Fernleaf |
|  | Covered with hairs |  | *B. textilis* | *B. distegia* |
|  | Covered with tomenta |  | *B. pachinensis* | *B. sinospinosa* |
|  | Covered with setae |  | *B. eutuldoides* var. *basistriata* | *B. arundinacea* |
|  | With a ring of tomenta below culm node |  | *B. sinospinosa* | *B. albolineata* |
|  | Color: green |  | *B. arundinacea* | *B. multiplex* cv. Alphonse-Karr |
|  | Color: green with yellow in concave |  | *B. albolineata* | *B. ventricosa* cv. Nana |
|  | Color: green and yellow |  | *B. multiplex* cv. Stripestem Fernleaf | *B. longispiculata* |
|  | Color: yellow with green in concave |  | N/A | *B. flexuosa* |
|  | Color: yellow with few green streak |  | *B. eutuldoides* var. *viridi-vittata* | *B. cerosissima* |
|  | Color: colorful streak |  | *B. eutuldoides* | *B. vulgaris* cv. Wamin |
|  | Internodes 0-30 cm long at 2 m high | Include 30 cm | *B. tuldoides* cv. Swolleninternode | *B. eutuldoides* |
|  | Internodes 30-50 cm long at 2 m high |  | *B. boniopsis* | *B. oldhamii / D. oldhamii* |
|  | Internodes over 50 cm long at 2 m high | Include 50 cm | *B. duriuscula* | *B. macrotis* |
|  | Internodes terete |  | *B. longispiculata* | *B. vulgaris* cv. Wamin |
|  | Sheath-node raised inconspicuously |  | *B. emeiensis / N. affinis* | *B. gibba* |
|  | Sheath-node raised slightly |  | *B. gibboides* | *B. prominens* |
|  | Sheath-node raised obviously |  | *B. lenta* | *B. duriuscula* |
|  | 0-3 cm in diam | Include 3 cm | *B. multiplex* var. *shimadae* | *B. pachinensis* var. *hirsutissima* |
|  | 3-8 cm in diam |  | *B. textilis* cv. Purpurascens | *B. multiplex* var. *riviereorum* |
|  | Over 8 cm in diam | Include 8 cm | *B. sinospinosa* | *B. xiashanensis* |
| Branch | With thorn |  | *B. mutabilis* | *B. gibboides* |
|  | With soft thorns |  | *B. eutuldoides* var.*basistriata* | *B. indigena* |
|  | With hard thorns |  | *B. macrotis* | *B. flexuosa* |
|  | Branching from lowest nodes 0-1.3 m above ground | Include 1.3 cm | *B. vulgaris* cv. Wamin | *B. textilis* |
|  | Branching from lowest nodes 1.3-2.6 m above ground |  | *B. vulgaris* cv. Vittata | *B. textilis* var. *gracilis* |
|  | Branching from lowest nodes over 2.6 m above ground | Include 2.6 cm | *B. pachinensis* var. *hirsutissima* | *B. pervariabilis* |
|  | Ultimate branchlets with 0-5 leaves |  | *B. xiashanensis* | *B. cornigera* |
|  | Ultimate branchlets with 6-9 leaves |  | *B. arundinacea* | *B. ventricosa* cv. Nana |
|  | Ultimate branchlets with over 10 leaves | Include 10 leaves | *B. tuldoides* cv. Swolleninternode | *B. multiplex* |
| Leaf | Length: 0-10 cm | Include 10 cm | *B. multiplex* var. *riviereorum* | *B. duriuscula* |
|  | Length: 10-20 cm |  | *B. pachinensis* | *B. eutuldoides* var. *basistriata* |
|  | Length: over 20 cm | Include 20 cm | *B. cerosissima* | *B. multiplex* cv. Fernleaf |
|  | Abaxially hairy |  | *B. macrotis* | *B. vulgaris* |
|  | Leaf base shape: wedgy |  | *B. cerosissima* | *B. textilis* cv. Purpurascens |
|  | Leaf base shape: olivary |  | *B. multiplex* cv. Stripestem Fernleaf | *B. chungii* |
| Culm sheath | Covered with hair |  | *B. textilis* | *B. ventricosa* cv. Nana |
|  | Covered with white powder |  | *B. surrecta* | *B. gibba* |
|  | Covered with spot |  | *B. arundinacea* | *B. boniopsis* |
|  | With a ring of tomenta below sheath scars |  | *B. flexuosa* | *B. gibboides* |
|  | Brim with hair |  | *B. distegia* | *B. vulgaris* |
|  | Length shorter than internodes |  | *B. eutuldoides* var. *basistriata* | *B. vulgaris* cv. Wamin |
|  | Length equal with internodes |  | *B. cornigera* | *B. duriuscula* |
|  | Length over than internodes |  | *B. tuldoides* cv. Swolleninternode | *B. oldhamii / D. oldhamii* |
|  | colorful streak |  | *B. longispiculata* | *B. pachinensis* var. *hirsutissima* |
| Sheath auricle | Length: inconspicuous |  | *B. multiplex* cv. Silverstripe | *B. mutabilis* |
|  | Length: below 1 cm long |  | *B. lenta* | *B. xiashanensis* |
|  | Length: 1-3 cm long |  | *B. eutuldoides* | *B. textilis* var. *gracilis* |
|  | Length: 3-5 cm long |  | *B. contracta* | *B. arundinacea* |
|  | Length: over 5 cm long |  | *B. oldhamii / D. oldhamii* | *B. macrotis* |
|  | Two auricles equally |  | *B. blumeana* | *B. textilis* var. *gracilis* |
|  | Two auricles length ratio value between 1.1-2 |  | *B. boniopsis* | *B. indigena* |
|  | Two auricles length ratio value between 2-3 |  | *B. gibboides* | *B. pachinensis* var. *hirsutissima* |
|  | Two auricles length ratio value between 3-4 |  | *B. eutuldoides* var. *basistriata* | *B. textilis* cv. Purpurascens |
|  | Two auricles length ratio value over 4 |  | *B. chungii* | *B. mutabilis* |
|  | Corrugated fold |  | *B. gibba* | *B. lenta* |
|  | Shape: half-circle | Only at 1.3 m above ground | *B. longispiculata* | *B. sinospinosa* |
|  | Shape: nearly cone | Only at 1.3 m above ground | *B. albolineata* | *B. pachinensis* |
|  | Shape: ovoid |  | *B. longispiculata* | *B. cerosissima* |
|  | Shape: long ovoid |  | *B. textilis* | *B. surrecta* |
|  | Shape: threadiness |  | *B. multiplex* cv. Silverstripe | *B. pervariabilis* |
|  | Oral setae length: 0-0.5 cm |  | *B. indigena* | *B. cornigera* |
|  | Oral setae length: 0.5-1.4 |  | *B. pachinensis* var. *hirsutissima* | *B. textilis* var. *gracilis* |
|  | Oral setae length: over 1.5 cm |  | N/A | *B. eutuldoides* |
|  | Oral setae straight |  | *B. lenta* | *B. eutuldoides* var. *viridi-vittata* |
|  | Auricles root and sheath blades separately |  | *B. vulgaris* cv. Vittata | *B. longispiculata* |
|  | Auricles root and sheath blades linked |  | *B. xiashanensis* | *B. mutabilis* |
|  | Auricles and sheath blades linked |  | *B. duriuscula* | *B. gibba* |
|  | No extension |  | *B. macrotis* | *B. tuldoides* cv. Swolleninternode |
|  | Auricles shouter than half extension |  | *B. oldhamii / D. oldhamii* | *B. albolineata* |
|  | Auricles longer than half extension |  | *B. eutuldoides* var. *basistriata* | *B. multiplex* |
| Sheath blade | Shape: triangular |  | *B. multiplex* cv. Alphonse-Karr | *B. longispiculata* |
|  | Shape: lanceolate |  | *B. chungii* var. *velutina* | *B. gibboides* |
|  | Erect (<90°) |  | *B. multiplex* var. *riviereorum* | *B. chungii* |
|  | Reflexed (ca. 90°) |  | *B. longispiculata* | *B. boniopsis* |
|  | Reflexed (>90°) |  | *B. chungii* | *B. mutabilis* |
|  | Straight without corrugation |  | *B. cerosissima* | *B. boniopsis* |
|  | Corrugation at 1/3 tip |  | N/A | *B. albolineata* |
|  | Corrugation at 1/3-1/2 tip |  | N/A | *B. distegia* |
|  | Corrugation at 1/1-2/3 tip |  | N/A | *B. textilis* cv. Purpurascens |
|  | Corrugation at the whole blade |  | *B. textilis* | *B. boniopsis* |
|  | Abaxially hairy |  | *B. blumeana* | *B. pachinensis* |
|  | Ventral hairy |  | *B. flexuosa* | *B. boniopsis* |
|  | Color: green |  | *B. gibboides* | *B. pachinensis* var. *hirsutissima* |
|  | Color: green and purple |  | N/A | *B. pervariabilis* |
|  | Color: colorful |  | *B. eutuldoides* var. *basistriata* | *B. vulgaris* cv. Wamin |
|  | Color: deadgress |  | N/A | *B. multiplex* |
|  | Tip shape: long | side over base less than 2 | *B. emeiensis / N. affinis* | *B. gibba* |
|  | Tip shape: acuminate | side over base equal to ca. 2 | *B. eutuldoides* | *B. vulgaris* |
|  | Tip shape: equilateral triangle | side over base equal to ca. 1 | *B. oldhamii / D. oldhamii* | *B. gibboides* |
|  | Culm sheath top length / sheath blade base length = 1-1.4 |  | *B. lenta* | *B. gibboides* |
|  | Culm sheath top length / sheath blade base length = 1.5-2.4 |  | *B. eutuldoides* var. *basistriata* | *B. multiplex* |
|  | Culm sheath top length / sheath blade base length = 2.5-3.4 |  | *B. longispiculata* | *B. multiplex* cv. Alphonse-Karr |
|  | Culm sheath top length / sheath blade base length = 3.5-4.4 |  | *B. chungii* | *B. multiplex* cv. Silverstripe |
|  | Culm sheath top length / sheath blade base length > 4.5 |  | *B. longispiculata* | *B. cerosissima* |
|  | Sheath clade length / culm sheath length < 1 |  | *B. textilis* | *B. tuldoides* cv. Swolleninternode |
|  | Sheath clade length / culm sheath length = 1 |  | *B. emeiensis / N. affinis* | *B. vulgaris* |
|  | Sheath clade length / culm sheath length > 1 |  | *B. vulgaris* cv. Wamin | *B. albolineata* |
| Sheath ligule | Length: 0-2 mm |  | *B. multiplex* cv. Fernleaf | *B. prominens* |
|  | Length: 2-4 mm |  | *B. flexuosa* | *B. cerosissima* |
|  | Length: over 4 mm |  | *B. blumeana* | *B. distegia* |
|  | Entire brim with hair (eyelash) |  | *B. macrotis* | *B. surrecta* |
|  | With slender eyelash |  | *B. albolineata* | *B. boniopsis* |
|  | With thick eyelash |  | *B. surrecta* | *B. indigena* |
|  | Eyelash 0-4 mm long | Include 4 mm | *B. textilis* | *B. sinospinosa* |
|  | Eyelash 4-8 mm long |  | *B. blumeana* | *B. ventricosa* cv. Nana |
|  | Eyelash over 8 mm long | Include 8 mm | *B. surrecta* | *B. macrotis* |
|  | Entire brim without hair |  | *B. boniopsis* | *B. multiplex* cv. Stripestem Fernleaf |
|  | Shape: sunken |  | *B. vulgaris* cv. Wamin | *B. pervariabilis* |
|  | Shape: flat |  | *B. duriuscula* | *B. textilis* var. *gracilis* |
|  | Shape: bulge |  | *B. lenta* | *B. gibboides* |

Characteristics of the culm sheath, sheath auricle, sheath blade, and sheath ligule were recorded at 1.3 and 2.0 m above ground, respectively, as two OTUs, except for the characteristics that have specific comments. Total characteristics comprised 186 OTUs.
