# Supplementary material for: Exploring phylogenetic relationships within the subgenera of Bambusa based on DNA barcodes and morphological characteristics
Source: Sci Rep. 2022 May 16;12:8018. doi: 10.1038/s41598-022-12094-8 (PMC9110708; doi:10.1038/s41598-022-12094-8)
Supplement: Supplementary file 2 — Supplementary Information 2. [file 41598_2022_12094_MOESM2_ESM.docx]

Table A2. The species list

| **No.** | | **Latin name** | | **Acquisition number** | |
| --- | --- | --- | --- | --- | --- |
| 1 | | *Bambusa albolineata* | | 2017080801 | |
| 2 | | *Bambusa arundinacea* | | 2017080802 | |
| 3 | | *Bambusa blumeana* | | 2017080803 | |
| 4 | | *Bambusa boniopsis* | | 2017080804 | |
| 5 | | *Bambusa cerosissima* | | 2017080805 | |
| 6 | | *Bambusa chungii* | | 2017080806 | |
| 7 | | *Bambusa chungii* var. *velutina* | | 2017080807 | |
| 8 | | *Bambusa cornigera* | | 2017080808 | |
| 9 | | *Bambusa contracta* | | 2017080809 | |
| 10 | | *Bambusa corniculata* | | 2017080810 | |
| 11 | | *Bambusa distegia* | | 2017080811 | |
| 12 | | *Bambusa dolichoclada* | | 2017080812 | |
| 13 | | *Bambusa duriuscula* | | 2017080813 | |
| 14 | | *Bambusa emeiensis/N. affinis* | | 2017080814 | |
| 15 | | *Bambusa eutuldoides* | | 2017080815 | |
| 16 | | *Bambusa eutuldoides* var.*basistriata* | | 2017080816 | |
| 17 | | *Bambusa eutuldoides* var. *viridi-vittata* | | 2017080817 | |
| 18 | | *Bambusa flexuosa* | | 2017080818 | |
| 19 | | *Bambusa gibba* | | 2017080819 | |
| 20 | | *Bambusa gibboides* | | 2017080820 | |
| 21 | | *Bambusa indigena* | | 2017080821 | |
| 22 | | *Bambusa lenta* | | 2017080822 | |
| 23 | | *Bambusa longispiculata* | | 2017080823 | |
| 24 | | *Bambusa macrotis* | | 2017080824 | |
| 25 | | *Bambusa multiplex* | | 2017080825 | |
| 26 | | *Bambusa multiplex* cv. Alphonse-Karr | | 2017080826 | |
| 27 | | *Bambusa multiplex* cv. Fernleaf | | 2017080827 | |
| 28 | | *Bambusa multiplex* cv. Silverstripe | | 2017080828 | |
| 29 | | *Bambusa multiplex* cv. Stripestem Fernleaf | | 2017080829 | |
| 30 | | *Bambusa multiplex* var*. riviereorum* | | 2017080830 | |
| 31 | | *Bambusa multiplex* var. *shimadae* | | 2017080901 | |
| 32 | | *Bambusa mutabilis* | | 2017080902 | |
| 33 | | *Bambusa pachinensis* | | 2017080903 | |
| 34 | | *Bambusa pachinensis* var. *hirsutissima* | | 2017080904 | |
| 35 | | *Bambusa pervariabilis* | | 2017080905 | |
| 36 | | *Bambusa prominens* | | 2017080906 | |
| 37 | | *Bambusa sinospinosa* | | 2017080907 | |
| 38 | | *Bambusa surrecta* | | 2017080908 | |
| 39 | | *Bambusa teres* | | 2017080909 | |
| 40 | | *Bambusa textilis* | | 2017080910 | |
| 41 | | *Bambusa textilis* var. *gracilis* | | 2017080911 | |
| 42 | | *Bambusa textilis* cv. Purpurascens | | 2017080912 | |
| 43 | | *Bambusa tuldoides* | | 2017080913 | |
| 44 | | *Bambusa tuldoides* cv. Swolleninternode | | 2017080914 | |
| 45 | | *Bambusa ventricosa* cv. Nana | | 2017080915 | |
| 46 | | *Bambusa vulgaris* | | 2017080916 | |
| 47 | | *Bambusa vulgaris* cv. Vittata | | 2017080917 | |
| 48 | | *Bambusa vulgaris* cv. Wamin | | 2017080918 | |
| 49 | | *Bambusa xiashanensis* | | 2017080919 | |
| 50 | | *Bambusa oldhamii / D. oldhamii* | | 2017080920 | |
| 51 | | *Dendrocalamus membranaceus* | | 2018070201 | |
| 52 | | *Dendrocalamus minor* | | 2018070202 | |
| 53 | | *Dendrocalamus minor* var. *amoenus* | | 2018070203 | |
| 54 | | *Drepanostachyum scandens* | | 2018070204 | |
| 55 | | *Indosasa shibataeoides* | | 2018070205 | |
| 56 | | *Melocanna baccifera* | | 2018070206 | |
| 57 | | *Neosinocalamus affinis* cv. Viridiflavus | | 2018070207 | |
| 58 | | *Oligostachyum lubricum* | | 2018070208 | |
| 59 | | *Phyllostachys heteroclada* | | 2018070209 | |
| 60 | | *Phyllostachys heterocycla* | | 2018070210 | |
| 61 | | *Phyllostachys violascens* | | 2018070211 | |
| 62 | | *Pseudosasa amabilis* | | 2018070212 | |
| 63 | | *Pseudosasa japonica* var. *tsutsumiana* | | 2018070213 | |
| 64 | | *Pleioblastus viridistriatus* | | 2018070214 | |
| 65 | | *Shibataea chinensis* cv. Aureo-striata | | 2018070215 | |
| 66 | | *Sinobambusa tootsik* var. *luteolo-albo-striata* | | 2018070216 | |

Nos. 1-50 were collected by Zou Yueguo in Hua'an Bamboo Botanical Garden. Nos. 51-66 were collected by Gu LiJian in Hangzhou Lin’an Taihuyuan Ornamental Bamboo Planting Garden.
